# Impacts of climate change adaptation options on soil functions: A review of European case‐studies

**DOI:** 10.1002/ldr.3006

**Published:** 2018-05-30

**Authors:** Ahmad Hamidov, Katharina Helming, Gianni Bellocchi, Waldemar Bojar, Tommy Dalgaard, Bhim Bahadur Ghaley, Christian Hoffmann, Ian Holman, Annelie Holzkämper, Dominika Krzeminska, Sigrun H. Kværnø, Heikki Lehtonen, Georg Niedrist, Lillian Øygarden, Pytrik Reidsma, Pier Paolo Roggero, Teodor Rusu, Cristina Santos, Giovanna Seddaiu, Eva Skarbøvik, Domenico Ventrella, Jacek Żarski, Martin Schönhart

**Affiliations:** ^1^ Leibniz Centre for Agricultural Landscape Research (ZALF) Eberswalder Straße 84 15374 Müncheberg Germany; ^2^ Tashkent Institute of Irrigation and Agricultural Mechanization Engineers (TIIAME) 39 Kary‐Niyaziy Street Tashkent 100000 Uzbekistan; ^3^ Faculty of Landscape Management and Nature Conservation University for Sustainable Development (HNEE) Schickler Straße 5 16225 Eberswalde Germany; ^4^ INRA, VetAgro Sup, UCA, Unité Mixte de Recherche sur Écosystème Prairial (UREP) 63000 Clermont‐Ferrand France; ^5^ Faculty of Management University of Science and Technology Fordońska 430 St. 85‐790 Bydgoszcz Poland; ^6^ Department of Agroecology Aarhus University Blichers Allé 20 DK‐8830 Tjele Denmark; ^7^ Department of Plant and Environmental Sciences, Faculty of Science University of Copenhagen Højbakkegård Allé 30 DK‐2630 Taastrup Denmark; ^8^ Institute for Regional Development European Academy of Bolzano Viale Druso 1 39100 Bolzano Italy; ^9^ Cranfield Water Science Institute Cranfield University Cranfield Bedford MK43 0AL UK; ^10^ Agroscope, Climate and Agriculture Group Reckenholzstrasse 191 8046 Zurich Switzerland; ^11^ Norwegian Institute of Bioeconomy Research, NIBIO Postbox 115 1431 Ås Norway; ^12^ Natural Resources Institute Finland (Luke) Latokartanonkaari 9 FI‐00790 Helsinki Finland; ^13^ Institute for Alpine Environment European Academy of Bolzano Viale Druso 1 39100 Bolzano Italy; ^14^ Plant Production Systems group Wageningen University and Research P.O. Box 430 6700 AK Wageningen The Netherlands; ^15^ Department of Agricultural Sciences University of Sassari viale Italia 39 07100 Sassari Italy; ^16^ Desertification Research Centre University of Sassari viale Italia 39 07100 Sassari Italy; ^17^ University of Agricultural Sciences and Veterinary Medicine Cluj‐Napoca Manastur Street 3‐5 400372 Cluj‐Napoca Romania; ^18^ IFAPA‐Centro Alameda del Obispo, Junta de Andalucía P.O. Box 3092 14080 Córdoba Spain; ^19^ Consiglio per la ricerca in agricoltura e l'analisi dell'economia agraria (CREA), Centro di ricerca Agricoltura e Ambiente (CREA‐AA) Via Celso Ulpiani 5 70125 Bari Italy; ^20^ Faculty of Agriculture and Biotechnology University of Science and Technology Bernardyńska St. 6 85029 Bydgoszcz Poland; ^21^ Department of Economics and Social Sciences University of Natural Resources and Life Sciences (BOKU) Feistmantelstraße 4 1180 Vienna Austria

**Keywords:** agricultural adaptation, DPSIR, regional case‐studies, soil degradation, Sustainable Development Goals

## Abstract

Soils are vital for supporting food security and other ecosystem services. Climate change can affect soil functions both directly and indirectly. Direct effects include temperature, precipitation, and moisture regime changes. Indirect effects include those that are induced by adaptations such as irrigation, crop rotation changes, and tillage practices. Although extensive knowledge is available on the direct effects, an understanding of the indirect effects of agricultural adaptation options is less complete. A review of 20 agricultural adaptation case‐studies across Europe was conducted to assess implications to soil threats and soil functions and the link to the Sustainable Development Goals (SDGs). The major findings are as follows: (a) adaptation options reflect local conditions; (b) reduced soil erosion threats and increased soil organic carbon are expected, although compaction may increase in some areas; (c) most adaptation options are anticipated to improve the soil functions of food and biomass production, soil organic carbon storage, and storing, filtering, transforming, and recycling capacities, whereas possible implications for soil biodiversity are largely unknown; and (d) the linkage between soil functions and the SDGs implies improvements to SDG 2 (achieving food security and promoting sustainable agriculture) and SDG 13 (taking action on climate change), whereas the relationship to SDG 15 (using terrestrial ecosystems sustainably) is largely unknown. The conclusion is drawn that agricultural adaptation options, even when focused on increasing yields, have the potential to outweigh the negative direct effects of climate change on soil degradation in many European regions.

## 1 INTRODUCTION

Soil systems are fundamental to sustainable development due to their multifunctional role in providing services including biomass production (food, feed, fibre, and fuel); habitats for living organisms and gene pools (biodiversity); cleaning of water and air; mitigation of greenhouse gas emissions; contributions to carbon (C) sequestration; buffering of precipitation extremes; and provisions to cultural, recreational, and human health assets (Coyle, Creamer, Schulte, O'Sullivan, & Jordan, 2016; Montanarella, 2015; Tóth et al., 2013). The effects of climate change are associated with increases in temperature (T) and extreme weather events such as heavy rainfall, droughts, frosts, storms, and rising sea levels in coastal areas. These effects may also increase the threats to soil such as soil erosion, soil compaction, reduced soil fertility, and lowered agricultural productivity, which ultimately deteriorate food security and environmental sustainability (Lal et al., 2011). These climate‐related risks raise major concerns regarding the future role of soils as a sustainable resource for food production.

Climate change can affect soil functions directly and indirectly. The direct effects include soil process changes in organic carbon transformations and nutrient cycling through altered moisture and T regimes in the soil or increased soil erosion rates due to an increased frequency of high‐intensity rainfall events. Blum (1993) was among the first to frame a systematic concept of linking soil processes via soil functions to services for the environment and society in Europe. Climate change and soil management can change the ability of soils to perform soil functions, which, for the sake of simplicity, the study calls changes in soil functions. Several studies have assessed the effects of climate change on soil functions (Coyle et al., 2016; Ostle, Levy, Evans, & Smith, 2009; Xiong et al., 2014). For instance, in organic‐rich soils in the UK, increased T and decreased soil moisture linked to warming or drought were observed to reduce the C storage capacity (Ostle et al., 2009).

The indirect effects of climate change on soil functions include those that are induced by climate change adaptation options. Agricultural management can mitigate climate change effects, for example, through increased soil organic carbon (SOC) sequestration (Haddaway et al., 2015). Farmers may implement adaptations as a result of multiple, intertwined driving forces, including market price changes, new technologies, and improved knowledge in combination with climate change (Reidsma et al., 2015b). Regarding European agriculture, several scenario studies have investigated agricultural adaptation options in response to climate change, including the introduction of irrigation regimes in drought‐prone areas, crop rotation changes, increased fertilization rates on cropland, amended soil tillage practices, and cultivation of melting permafrost soils (Mandryk, Reidsma, & van Ittersum, 2017; Schönhart, Schauppenlehner, Kuttner, Kirchner, & Schmid, 2016; Ventrella, Charfeddine, Moriondo, Rinaldi, & Bindi, 2012a).

Although ample knowledge is available for the direct effects (although the interactions are not completely understood), evidence of the indirect effects of agricultural adaptation options on soil functions is more scattered and difficult to derive experimentally because it depends on an uncertain future climate and corresponding adaptation. However, the anticipation of development pathway impacts is a precondition for decision‐making.

Although farm management concerns the local field level, the multiple soil functions need to be maintained and improved at higher spatial aggregates to achieve the Sustainable Development Goals (SDGs) formulated by the United Nations agenda 2030. Montanarella and Alva (2015) assessed soil functions as being particularly relevant for three of the 17 SDGs, namely, SDGs 2 (achieving food security and promoting sustainable agriculture), 13 (taking actions on climate change), and 15 (using terrestrial ecosystems sustainably, reversing land degradation, and halting biodiversity loss).

The objective of this paper was to review case‐studies on future adaptation options in European regions for their information on how adaptations may affect soil functions and what that means in the context of the SDGs. Taking current climate systems and management practices as counterfactuals, the cases were used to assess how future climate change in combination with adaptation options may impact European soils. The regional case‐studies resulted from the European Joint Programming Initiative on Agriculture, Climate Change, and Food Security (FACCE‐JPI) knowledge hub MACSUR (Modelling European Agriculture with Climate Change for Food Security; http://www.macsur.eu). MACSUR brought together researchers across Europe to improve the understanding of climate change impacts and adaptation potentials on European agriculture.

## 2 MATERIALS AND METHODS

### 2.1 Study area and climate

**Figure 1:**
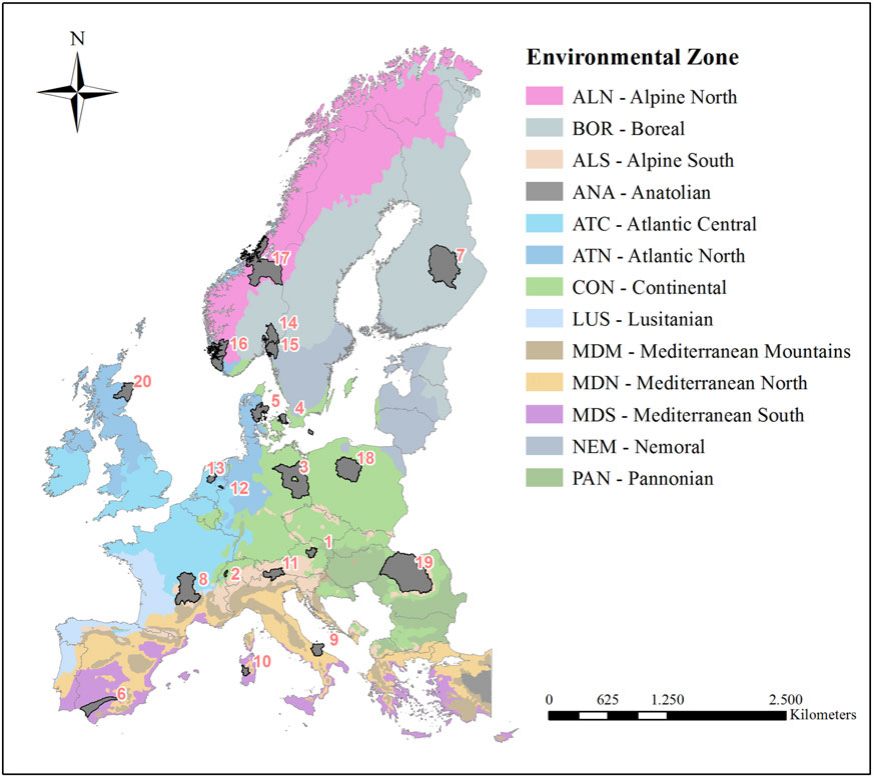
Location of the 20 case‐study areas and their environmental zones in Europe as classified by Metzger et al. (2005): 1—Mostviertel (AUT), 2—Broye (CH), 3—Brandenburg (DE), 4—Hovedstaden (DK), 5—Norsminde (DK), 6—Guadalquivir Valley (ES), 7—North Savo (FI), 8—Massif Central (FR), 9—Foggia (IT), 10—Oristanese (IT), 11—South Tyrol (IT), 12—Baakse Beek (NL), 13—Flevoland (NL), 14—Hobøl, Østfold (NO), 15—Jæren, Rogaland (NO), 16—Lowland Trøndelag (NO), 17—Romerike Akershus (NO), 18—Kujawsko‐Pomorskie (PL), 19—Transylvanian Plain (RO), and 20—NE Scotland (UK) [Colour figure can be viewed at http://wileyonlinelibrary.com]

Climate change adaptation options and resulting soil impacts are likely to be diverse across Europe due to heterogeneous biophysical and socio‐economic production conditions. Additionally, research design likely determines conclusions on adaptation options and their impacts in a region. To tackle both bio‐physical and socio‐economic dimensions, 20 case‐studies across Europe were assessed at the NUTS 2/3 level (Figure 1). Each case‐study undertook an integrated assessment with quantitative tools (e.g., scenario modelling) or qualitative, stakeholder inclusive tools or a combination of both. Published results from case‐studies were compiled and further substantiated with information from 23 involved scientists—most of them co‐authors of this article—via a semi‐structured questionnaire (Appendix S1). This led to a unique data set that reflects the impacts of adaptation options on soils across Europe. The 20 case‐studies represent 13 European countries and cover 11 of the 13 major environmental zones of Europe (Metzger, Bunce, Jongman, Mücher, & Watkins, 2005). This classification represents the environmental heterogeneity of Europe and utilizes European ecological data sets for climate, geomorphology, geology and soil, habitats, and vegetation. The two zones not presented in the sample are Anatolia and Lusitania.

To classify the case‐studies in terms of soil types, the World Reference Base for Soil Resources (FAO, 2006) was used. The 20 case‐study areas cover the 15 most common arable soil types of the 32 World Reference Base types (Table 1). Table 1 also lists the features of climate change scenarios that are relevant to agricultural production, land use and farming systems, methods employed to obtain the results, and key publications for each of the case‐studies. Regarding the assessment methods, most studies (17 out of 20) modelled the effects of alternative adaptation management options under climate change on yields and environmental impacts. Such adaptation options were identified by means of stakeholder interaction with regional farmers or extension services in 14 cases and by researchers in the other cases. Therefore, the adaptation options that were regarded as the most suitable by farmers could be identified. Three case‐studies simulated changing climatic conditions by employing field experiments at different locations for studying adaptation options (e.g., crop rotation and no tillage).

**Table 1:** Characteristics of the 20 case‐studies

Case‐studies (name of region and country)	Climate change characteristics, most relevant for agriculture			Land use/ farming system	Main soil types. WRB classification	Dominant topsoil texture	Assessment method	References
	Increased T	Severe rainfall events	Drought events					
Mostviertel (AUT)	X	X		Arable, livestock	Luvisols	Sandy silt, loamy silt	Modelling, stakeholder interaction	Schönhart et al. (2016)
Broye (CH)	X	X	X	Arable, some irrigated, permanent crops, pasture	Cambisols	Sandy loam, loam	Modelling, stakeholder interaction	Klein et al. (2013)
Brandenburg (DE)			X	Arable, some irrigated	Luvisols, fluvisols, cambisols	Loamy sand	Modelling, GIS, stakeholder interaction	Gutzler et al. (2015)
Hovedstaden (DK)	X	X		Arable	Calcisols	Sandy clay loam, clay loam	Field experiments	Ghaley, Vesterdal, and Porter (2014)
Norsminde (DK)	X	X	X	Arable	Luvisols	Clay, loam, sand	Modelling, GIS, stakeholder interaction	Odgaard et al. (2011)
Guadalquivir Valley (ES)	X	X	X	Arable, rainfed cropping, some irrigated	Vertisols, cambisols, regosols	Clay, silt	Modelling	Gabaldón‐Leal et al. (2015)
North Savo (FI)	X	X		Arable, rotational grasslands, livestock	Albeluvisols, podzols, luvisols, histosols	Sand, silt, clay, peat	Modelling, stakeholder interaction	Huttunen et al. (2015)
Massif Central (FR)	X			Arable, some irrigated, permanent crops	Cambisols	Silt	Modelling, stakeholder interaction	Klumpp et al. (2011)
Foggia (IT)	X	X	X	Arable, rainfed cropping, irrigation	Luvisols, cambisols, vertisols	Clay, silty clay	Modelling	Ventrella, Giglio, et al. (2012b)
Oristanese (IT)	X	X	X	Arable, some irrigated	Fluvisols, cambisols, luvisols	Clay, sands	Modelling, stakeholder interaction	Dono et al. (2016)
South Tyrol (IT)	X	X		Permanent crops	Cambisols	Alluvial sandy loam	Field experiments	Thalheimer (2006)
Baakse Beek (NL)	X	X	X	Livestock, arable	Cambisols, luvisols, podzols	Sand	Modelling, stakeholder interaction	Reidsma et al. (2015a)
Flevoland (NL)	X	X	X	Arable, some irrigated	Fluvisols	Marine clay	Modelling, stakeholder interaction	Mandryk et al. (2017)
Hobøl, Østfold (NO)	X	X	X	Arable, permanent crops	Albeluvisols, stagnosols, anthropic regosols/technosols	Silty clay loam, silt loam, sand, silt	Modelling, stakeholder interaction	Skarbøvik and Bechmann (2010)
Jæren, Rogaland (NO)	X	X		Arable, permanent crops, livestock	Umbrisols, gleysols, histosols, stagnosols	Loamy sand, organic	Stakeholder interaction	Hauken and Kværnø (2013)
Lowland Trøndelag (NO)	X	X		Arable, permanent crops, livestock	Stagnosols, cambisols, albeluvisols, anthropic regosols/technosols	Silty clay loam, silt loam, sand	Stakeholder interaction	Hauken and Kværnø (2013)
Romerike Akershus (NO)	X	X	X	Arable, permanent crops, livestock	Stagnosols, cambisols, albeluvisols, anthropic regosols/technosols	Silty clay loam, silt loam, sand, silt	Stakeholder interaction, field experiments	Deelstra, Øygarden, Blankenberg, and Olav Eggestad (2011)
Kujawsko‐Pomorskie (PL)		X	X	Arable, some irrigated	Luvisols, phaeozems	Loamy sand, clay	Stakeholder interaction, field experiments	Bojar et al. (2014)
Transylvanian Plain (RO)	X	X	X	Arable, permanent crops, pasture, livestock	Chernozems, phaeozems, luvisols	Silty clay, loam	Field experiments	Rusu et al. (2017)
NE Scotland (UK)	X	X		Arable, pasture, livestock	Cambisols, podzols	Medium clay	Modelling	Holman et al. (2016)

*Note*. GIS = Geographic Information System; T = temperature; WRB = World Reference Base.

### 2.2 Analytical framework

**Figure 2:**
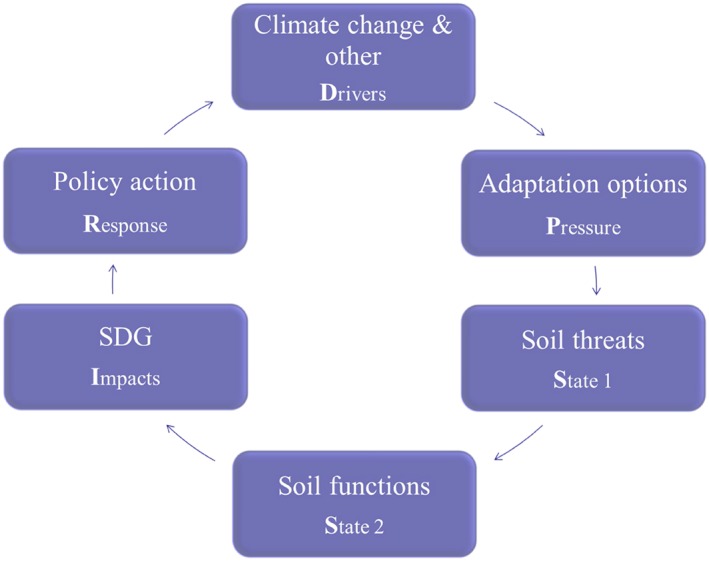
Analytical chain of the study applied to the Driver–Pressure–State–Impact–Response framework. SDG = Sustainable Development Goal Source: Adapted from Gabrielsen and Bosch (2003) [Colour figure can be viewed at http://wileyonlinelibrary.com]

The Driver–Pressure–State–Impact–Response framework was used to study the impacts of climate change adaptation options on the soil functions and SDGs (Figure 2). The framework conceptualizes complex sustainability challenges and provides insight into the relationships between the environment and human beings (Gabrielsen & Bosch, 2003). It links the emergence of climate change (Drivers of change) and its impacts on natural and human systems to decision makers (farmers) who adopt new management practices (Pressures), which can lead to soil threats (State 1) and altered soil functions (State 2). Subsequently, the SDG targets (Impact) can be affected. As a result, policy action (Response) may be required (not covered in the present study). Adaptation options, soil threats, and soil functions are understood as dynamic processes over time, such that the ‘States’ in the Driver–Pressure–State–Impact–Response framework represent dynamic biophysical indicators and human practices.

Adaptation options can be triggered by climate change. However, in reality, this driver is intertwined with other factors such as market conditions, technological development, farmer perceptions, and policy interventions (Mitter, Schönhart, Larcher, & Schmid, 2018; Techen & Helming, 2017). All case‐studies assessed climate change adaptation but in different scenario contexts. For the sake of comparability, only those scenarios and adaptation options were included in the review that had been developed from a farming system perspective intended to maintain farm profitability and improve yield level and stability. Other adaptation options focusing primarily on environmental (e.g.,reduced nutrient leaching) and/or social (e.g., employment, health, and culture) objectives (Mandryk, Reidsma, Kanellopoulos, Groot, & van Ittersum, 2014) were not included. The current situation of management practices and climate conditions is the counterfactual to which scenarios of future climate and management situations were assessed. However, in reality, transition is already occurring, and the adoption of adaptation practices can already be observed at individual farms in some cases (e.g., in North Savo, FI).

### 2.3 Characteristics of soil threats and soil functions

The European Commission's (2002) report lists seven major threats that cause soil degradation in Europe: *soil erosion*, *decline in SOC*, *compaction*, *decline in soil biodiversity*, *salinization*, *contamination*, and *sealing*. Because the study focuses on agricultural soil management, only the first five soil threats were considered. Soil contamination and soil sealing were excluded because the first is by definition associated with industrial, mainly point‐source pollution, whereas the latter refers to taking land out of production (European Commission, 2002).

Soils provide numerous functions to society. The European Commission (2006) lists seven key functions: *food and biomass production*; *storing*, *filtering, transforming*, *and recycling water and nutrients*; *habitat and gene pool*; *SOC pool*; *providing raw materials*; *serving as physical and cultural environment for mankind*; and *storing the geological and archaeological heritage*. In this study, focus was laid on the first four functions (Table 2), which are most relevant to agricultural land use (Schulte et al., 2014). The concept of soil functions was introduced in the Thematic Strategy for Soil Protection (European Commission, 2006), although it has not resulted in a legal implementation of soil conservation measures. Soil functions connect the physical, chemical, and biological processes in the soil system with the provision of benefits to society (Glæsner, Helming, & de Vries, 2014). Agricultural management affects the performance of soil functions in close interaction with geophysical site conditions. The optimization of one of the functions is often to the disadvantage of others. The assessment presents aggregated impacts of one to several adaptation options on soil threats and functions (Table 3).

**Table 2:** Soil functions and the linkage to the SDGs as classified by Montanarella and Alva (2015)

Soil functions	Linkage to the SDGs
Food and biomass production	Link to agriculture and biomass provision for food, fibre, energy: SDG 2 ‘Food security and sustainable agriculture’
Storing, filtering, transforming, and recycling	Link to water quality, nutrients, flood control, microclimate, ecosystem resilience, detoxification: SDG 15 ‘Terrestrial ecosystems: land degradation and biodiversity’
Habitat and gene pool	Link to biodiversity: SDG 15 ‘Terrestrial ecosystems: land degradation and biodiversity’
Soil organic carbon pool	Link to climate change mitigation: SDG 13 ‘Climate action’

*Note*. SDGs = Sustainable Development Goals.

**Table 3:** Expected agricultural adaptation options and anticipated impacts on soil threats and soil functions in the 20 case studies

Case‐Studies	Adaptation options					Soil threats^a^					Soil functions^a^			
	Crops and crop rotation	Tillage	Irrigation/drainage	Fertilization	Share of arable land	Soil erosion	SOC decline	Compaction	Biodiversity	Salinization	Food and biomass production	Storing, filtering, transforming, recycling	Habitat and gene pool	SOC pool
Mostviertel (AUT)	More wheat	Increase conservation tillage	Small increase in irrigation extent	Increase amount	Increase cropland, reduce grassland	−	+	+			+	+		+
Broye (CH)	More rainfed winter barley	Increase conservation tillage	Increase irrigation for key crops		Increase grassland, reduce cropland	+					+	+		
Brandenburg (DE)	More maize		Introduce irrigation for key crops	Increase amount		−		−			+			
Hovedstaden (DK)	Diversify crop rotation	Minimize tillage traffic				+	+		+		+	+	+	+
Norsminde (DK)	More catch crops and grass, less maize	Increase conservation tillage	Control drainage	Increase amount	Reduced area in rotation	+	+	+	+		+	+	+	+
Guadalquivir Valley (ES)		Increase conservation tillage	Increase irrigation efficiency			+	+	−	+	−	+	+		+
North Savo (FI)	More clover, oilseed		Improve drainage system	Increase amount and efficiency		+	+	+	+		+	+	+	+
Massif Central (FR)	More maize					+	+				+	+		+
Foggia (IT)	More winter wheat, tomato		Increase irrigation efficiency	Increase efficiency		+	+			−	+	+		+
Oristanese (IT)	More grain, forage	Increase conservation tillage	Increase in irrigation areas and efficiency	Increase efficiency	Increase cropland		+		+		+	+	+	+
South Tyrol (IT)	Same crop but adapted varieties		Increase irrigation efficiency					−	−		+		−	
Baakse Beek (NL)	More maize, potato			Reduce amount	Increase cropland, reduce grassland		−		−		+	−	−	−
Flevoland (NL)	More winter wheat		Increase irrigation efficiency				+	+			+	+		+
Hobøl, Østfold (NO)	More forage	Increase conservation tillage	Improve drainage system		Increase grassland, reduce cropland	+	+	+	+		−	+	+	+
Jæren, Rogaland (NO)			Improve drainage system		Increase grassland, reduce cropland		+	−			−	−		+
Lowland Trøndelag (NO)			Improve drainage system		Increase grassland, reduce cropland	+	+	−			−	−		+
Romerike Akershus (NO)	More forage	Increase conservation tillage	Improve irrigation system		Increase grassland, reduce cropland	+	+	+			−	+		+
Kujawsko‐Pomorskie (PL)	More cereals, maize, rape	Increase conservation tillage	Increase irrigation efficiency	Increase amount		+	+	−	+		+	+		+
Transylvanian Plain (RO)	More maize, soybean, wheat	Increase conservation tillage	Introduce irrigation for key crops	Apply organic fertilizers		+	+	−	+		+	+	+	+
NE Scotland (UK)					Increase cropland, intensify grassland	−	−	−			+	−		−

*Note*. SOC = soil organic carbon.

a (+) Positive impact = reduced soil threats and improved soil functions; (−) negative impact = increased soil threats and decreased soil functions.

### 2.4 Relevance of soil functions for realizing the SDGs

In 2015, the United Nations member countries adopted the agenda 2030 with its 17 SDGs. Although not explicit in the 17 SDG guidelines, the ability of soils to perform their functions plays an important role in meeting specific goals (Keesstra et al., 2016). The review of case‐studies was used to examine the potential of supporting the SDGs in the European context through links with soil functions (Montanarella & Alva, 2015; Table 2).

## 3 RESULTS AND DISCUSSION

The results indicate that all case‐studies considered soil degradation, although they all had other primary research objectives (e.g., yields, profitability, and greenhouse gas emissions). This confirms the high awareness of soil degradation issues in agricultural climate change research. In general, the adaptation options under climate change conditions seem to have positive impacts on soils (Table 3). Five main groups of agricultural adaptation options could be distinguished: introduction of new crops and crop rotation changes; alteration of the intensity of tillage practices; implementation of irrigation and drainage systems; optimization of fertilization; and change of arable land to grassland or vice versa. The potential soil threats of adaptation options and impacts on soil functions are presented in Table 3. A positive impact (+) indicates reduced soil threats and improved soil functions. A negative impact (−) indicates increased soil degradation risks and decreased soil functions. Due to the aggregation of one to several simultaneously assessed adaptation options, the combined effects on soil functions are provided for each case‐study.

### 3.1 Impacts of adaptation options on soil threats

**Figure 3:**
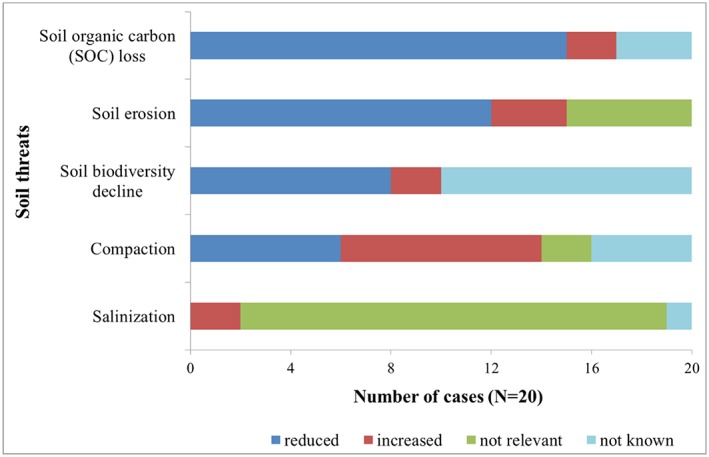
Anticipated impacts of agricultural adaptation options on soil threats [Colour figure can be viewed at http://wileyonlinelibrary.com]

The study shows that adaptation options under climate change scenarios reduced SOC losses in 75% of the cases examined (Figure 3). For example, farmers and extension experts in the North Savo case (FI) are already worried about wet conditions in winter and more frequent heavy rains as well as wet conditions during the harvest periods, which affect crop yields, nutrient leaching, and erosion. In response, modified crop rotations, including the use of deep‐rooted crops (i.e., clover and oilseed), have been proposed by local scientists (Huttunen et al., 2015; Peltonen‐Sainio et al., 2016). An expert from the region anticipates that these changes may maintain or even improve the SOC levels and water retention. For the case‐study of Foggia (IT), adopting 2‐ or 3‐year crop rotations (based on winter wheat and tomato) under future conditions similar to a climate model realization of the IPCC A2 climate emission scenario led to an increase in SOC by approximately 10% of the SOC content of the current system that is based on continuous wheat (Ventrella et al., 2012b).

The SOC levels were expected to decrease in only two cases (10%) as a result of implementing adaptation options. For example, using the CLIMSAVE Integrated Assessment Platform, Holman, Harrison, and Metzger (2016) identified adaptation options for NE Scotland (UK). The options included an expansion of the agricultural area and conversion of extensive permanent grassland to ley grassland and arable land due to expected increases in T and reduced summer wetness limitations by 2050. These measures would likely lead to a loss of SOC in the area. Three case‐studies (15%) did not analyse SOC changes.

Twelve studies (60%) anticipated a reduced potential risk of soil erosion due to implementation of adaptation measures, including improved crop rotations, permanent soil cover by crop residues, and minimum tillage or zero tillage.

Although adaptation options are anticipated to reduce many soil threats in most cases across Europe, there are concerns regarding the likely increase in soil compaction (approximately 40%). Soil compaction is a common problem worldwide. It affects plant root development and reduces water retention capacity; it can also lower crop yields (D'Or & Destain, 2016). With the increase in total irrigated cropland and more intensive use of agricultural machinery, the risk of soil compaction may increase. For Brandenburg (DE), Gutzler et al. (2015) identified the irrigation of key crops, such as wheat, rye, maize, and sugar beet, as an agricultural adaptation strategy to cope with climate change (e.g., less rainfall in summer and more in winter) and to increase crop productivity. However, irrigation and the use of heavy machinery may increase the risk of soil compaction in the area. Thus, an appropriate use of agricultural machinery (e.g., low pressure and wide tires) is one effective measure against compaction (Prager et al., 2011). In Flevoland (NL), some farmers are concerned about SOC loss and soil compaction and therefore intend to replace root crops with wheat. However, if they were only interested in profits, the area of root crops such as potatoes would likely increase (Mandryk et al., 2017).

The results further show that little knowledge or awareness is currently available among agricultural researchers regarding the influence of climate change and adaptation on soil biodiversity, although the decline in soil biodiversity has been reported as the key future threat (McBratney, Field, & Koch, 2014). Although eight cases anticipated positive and two cases anticipated negative impacts on biodiversity, 10 cases (50%) did not consider soil biodiversity.

Most of the case‐studies reported that the risk of salinity is limited, at least in the medium term, due to their locations in northern and western parts of Europe. Salinity issues are more prominent in the southern and eastern parts of Europe, such as in the Mediterranean climate region (Zalidis, Stamatiadis, Takavakoglou, Eskridge, & Misopolinos, 2002), where the annual water balance may become negative. In the case of the Guadalquivir Valley (ES), increased irrigation using reclaimed wastewater might create environmental problems due to increased soil salinity accumulation. Studies carried out in Almería (southern Spain) showed that irrigation with nutrient enriched disinfected urban wastewater can result in low macronutrient absorption efficiency and high soil salinity (Segura, Contreras París, Plaza, & Lao, 2012).

### 3.2 Impacts of adaptation options on soil functions

**Figure 4:**
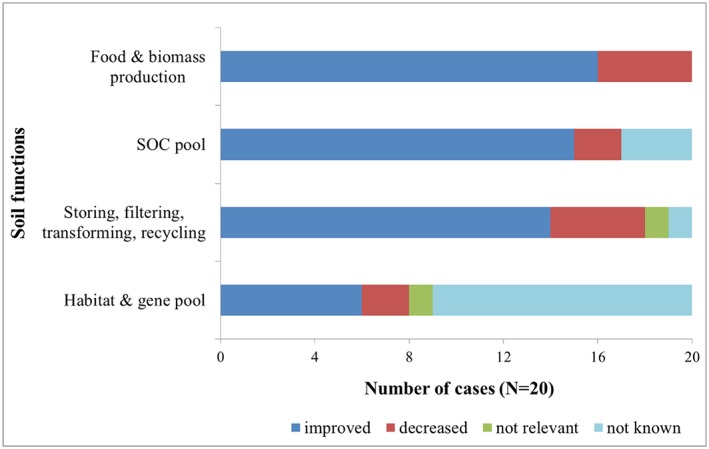
Anticipated impacts of agricultural adaptation options on soil functions. SOC = soil organic carbon [Colour figure can be viewed at http://wileyonlinelibrary.com]

In addition to reducing soil threats, most of the adaptation options were found to have positive effects on some soil functions (Figure 4). Adaptation options were expected to increase agricultural food and biomass production in 80% of the case‐studies. This finding reveals that the integration of climate change adaptation and yield increase was plausible for the time range of the studies (i.e., the years 2025–2100). In the example of Oristanese (IT), decreased rainfall in the spring and more frequent and extreme droughts are expected as part of climate change. Adaptation of crop varieties/hybrids and improved organic fertilizer use and management have been proposed to offset such climate change challenges when irrigation water is available (Dono et al., 2016), which may result in increased crop and biomass production due to the extended growing season, the CO_2_ fertilization, and the effect of milder winters on irrigated autumn–spring hay crops.

Increased biomass production accompanied an expected increase in SOC in 11 of the 16 cases. The results highlight that adaptation options such as reduced tillage, establishment of cover crops, and manuring have the possibility to maintain or even increase the SOC content. For example, Schönhart et al. (2016) illustrated the positive impacts of reduced tillage on the SOC levels for Mostviertel (AUT) based on integrated modelling.

The storing, filtering, transforming, and recycling functions of soils were also found to be positively impacted by the adaptation options in 70% of the case‐studies. For example, in the Broye (CH) case‐study, increasing irrigation resulted in a denser and more permanent crop cover throughout the year and therefore helped to maintain agricultural productivity and to reduce nutrient losses through leaching or soil loss through water erosion. Furthermore, it was found that both conservation soil management and an increase in the share of winter crops can contribute to a reduction in soil loss by providing soil coverage, particularly during the periods of the year with the most intense rainfall events (Klein, Holzkämper, Calanca, & Fuhrer, 2014).

Similar to the results of soil threats, the impacts on the function of soils as a habitat and gene pool are largely unknown. Of the 20 case‐studies, only six (30%) addressed the impacts of agricultural adaptation on soil biodiversity. The obvious ignorance of soil biodiversity issues in most of the case‐studies is a mismatch with the emerging knowledge of the important functional role of soil organisms for soil processes (Cluzeau et al., 2012). This is a clear knowledge gap that must be addressed in the future. Among the few cases addressing biodiversity, Odgaard, Bøcher, Dalgaard, and Svenning (2011) proposed adaptation, including changing crop rotations (e.g., reduced maize area) for Norsminde (DK). Increasing drainage and extending buffer zones along water courses (Christen & Dalgaard, 2013) can be responses to more extreme weather events. Local experts in Norsminde expect positive impacts on habitats with larger and perhaps more diverse gene pools. In general, in Denmark, there is a trend towards more organic farming, which will ultimately promote soil biodiversity.

### 3.3 Progress towards the SDGs

The adaptation options represented in the case‐studies potentially support the achievement of SDGs. Adaptation in most of the case studies likely supports SDGs 2 and 13, whereas the impacts on SDG 15 appear uncertain and depend on the regional context and the choice of adaptation options. Most case‐studies are largely based on modelling and experts' expectations of possible effects of future management and less on measured empirical evidence, which increases uncertainties of soil biodiversity effects due to climate change adaptation. However, with respect to SDGs 2 and 13, several climate change adaptation options are already practised on farms in order to increase resilience to harmful weather events (e.g., Mitter et al., 2018), which increases confidence. For example, some evidence has been found for effects on crop yields and soil functions under conditions of elevated temperatures, rainfall, or extreme events (Peltonen‐Sainio et al., 2016), which are most likely becoming more frequent due to climate change in some European regions. Other adaptation options, such as more diversified land use at the farm level suggested by Peltonen‐Sainio et al. (2016), require further empirical evidence.

Although the contribution to SDG 2 through increased food and biomass production in many areas of Europe is in line with other model results on climate change adaptation (Ergon et al., 2018; Gabaldón‐Leal et al., 2015; Klein, Holzkämper, Calanca, Seppelt, & Fuhrer, 2013; Klumpp, Tallec, Guix, & Soussana, 2011), less evidence is available to validate findings on the other soil functions, which are more important for SDGs 13 and 15. Further uncertainty results from the huge knowledge gap on the potential and adoption rates of emerging technologies in agriculture and on process interactions between climate change, soil management, and soil functions. Detailed, integrated case‐studies of climate and management changes are required to verify which adaptation options perform best to promote sustainable development in a particular regional context and how their adoption can be supported.

## 4 SUMMARY AND CONCLUSIONS

Climate change is a major threat that could lead to a decline in agricultural production in many regions of the world. Adaptation is important to manage the risks and utilize the benefits from climate change. However, when the primary aim is to increase food production, soils and ecosystem services may be adversely affected. Thus, understanding the possible future impacts of agricultural adaptation options for addressing potential risks of soil degradation is vital.

The results of this study provide some clear general insights. They show that adaptation options are expected to reduce the threats of soil erosion and declining SOC in most cases. Soil compaction remains a major threat. Little knowledge is available regarding the decline in soil biodiversity. Therefore, future research should focus on these shortcomings. Furthermore, the adaptation options reveal generally positive effects on the soil functions of food and biomass production, C sequestration in soil, and improvements in storing, filtering, transforming, and recycling capacities. Impacts on soil microorganisms and soil fauna are poorly understood. The results suggest that anticipated climate change adaptation options in agriculture have the potential to offset some of the deteriorating impacts of climate change on soil functions if farmers implement them based on the best available knowledge. In addition, the linkage between soil functions and the SDGs indicates a positive contribution to achieving SDGs 2 (achieving food security and promoting sustainable agriculture) and 13 (taking actions on climate change), whereas a clear signal regarding impacts on SDG 15 (using terrestrial ecosystems sustainably) could not be identified.

Finally, this study demonstrated that despite the broad range of local contexts and farming systems assessed in the 20 case‐studies across Europe, it is possible to identify converging win–win policies that are able to support adaptation options that could, at the same time, minimize soil threats and enhance multiple soil functions. However, more studies are needed in the future to support this ambition given the uncertainties inherent to climate change, its implications for long‐term soil process dynamics, interactions with agricultural practices, and the multiple interacting factors affecting the consequences of adaptation options as well as the market, technology, and policy changes for soils.

## Supporting information

Data S1 A semi‐structured interview format
