# Supplementary material for: Impacts of climate change adaptation options on soil functions: A review of European case‐studies
Source: Land Degrad Dev. 2018 May 30;29(8):2378–89. doi: 10.1002/ldr.3006 (PMC6199005; doi:10.1002/ldr.3006)
Supplement: Supplementary file 1 — Data S1 A semi‐structured interview format [file LDR-29-2378-s001.doc]

**Supplementary Appendix S1**

Appendix S1 A semi-structured interview format

***General information on case study:***

| Region name: |  | | | NUTS 2/3 code: | | | |
| --- | --- | --- | --- | --- | --- | --- | --- |
| Responsible person: | Name: | | | Email: | | | |
| Project origin: | MACSUR: yes/no | | | Other: | | | |
| Interested in joining publication: | yes/no | | | | | | |
| Available results: | Final/preliminary/conceptual | | | | | | |
| Methods used for adaptation assessment: | Model(s) used | | | Other methods | | | |
| References: |  | | | | | | |
| ***Climate change scenarios and adaptation options:*** | | | | | | | |
| Specifications of drivers of climate change (please specify): | e.g. increased rainfall variability and severe rainfall events  e.g. more frequent droughts | | | | | | |
| Key agricultural adaptation options (please specify): | farming system level (e.g. change in crop rotation) catchment scale (e.g. collective water or fertilizer management) district scale (e.g. synergies between rainfed and irrigated districts) | | | | | | |
| ***Anticipated impacts on soil threats and soil functions:*** | | | | | | | |
| Key soil threats in the case study (please describe): | Erosion | Compaction | Salinization | | Organic matter loss | Biodiversity loss | Other |
| Key soil functions in the case study (please describe): | Food and biomass | Storing, filtering, transforming, recycling | Habitat and gene pool | | Carbon pool | Other | |

***Guiding questions:***

- In your case study area, what are specifications of climate change with regards to natural conditions of agricultural soil management?
- What management practices stakeholders adapt/ed in addressing the climate change?
- What is the impact of your management practices on soil threats (positive/negative) and on soil functions (positive/negative)?
